# Promoting a Smartphone-Free Bedroom Environment to Enhance Sleep Quality among University Students: A Cross-Sectional Study from Saudi Arabia

**DOI:** 10.12669/pjms.41.5.10755

**Published:** 2025-05

**Authors:** Manal Almalki, Manal Abasher Mohamed, Ali M. Alzahrani

**Affiliations:** 1 Manal Almalki Department of Public Health, College of Nursing and Health Sciences, Jazan University, Jazan, Saudi Arabia; 2 Manal Abasher Mohamed Department of Public Health, College of Nursing and Health Sciences, Jazan University, Jazan, Saudi Arabia; 3 Ali M. Alzahrani Department of Health Administration and Hospitals, College of Public Health and Health Informatics, Umm Al-Qura University, Makkah, Saudi Arabia

**Keywords:** PSQI, Smartphone usage, Sleep quality, Undergraduate students, Sleep hygiene

## Abstract

**Objective:**

This study investigates the impact of smartphone usage habits on sleep quality among undergraduate students at Jazan University, Saudi Arabia.

**Methods:**

A cross-sectional study was conducted between April and May 2023 at Jazan University, Saudi Arabia. An online questionnaire included three sections: demographic characteristics, smartphone usage habits, and the Pittsburgh Sleep Quality Index (PSQI) for assessing sleep quality. Descriptive analysis and inferential tests were utilized.

**Results:**

Among 1,153 participants, the average PSQI score of 8.19, indicating poor sleep quality. Nearly three-quarters (73.46%) of participants used smartphones for over six hours daily. Regression analysis revealed three significant predictors of poor sleep: smartphone use in the evening, waking up frequently to check phones, and spending 1-2 hours on a smartphone at bedtime. Smartphone use for two hours or more before bedtime was linked to prolonged sleep latency (31.21%), frequent sleep disturbances (16.67%), increased reliance on sleep medications (4.96%), and notable daytime dysfunction (4.96%). Waking up twice or more to check smartphones had the strongest association with poor sleep quality.

**Conclusion:**

High smartphone use is a risk factor for poor sleep quality among university students. Promoting a smartphone-free bedroom environment is suggested as an effective strategy to enhance sleep hygiene and reduce frequent sleep interruptions.

## INTRODUCTION

Sleep issues are a significant concern among university students worldwide. A study in China found that 26.64% of 8,457 undergraduate students had poor sleep quality, with older students and those in higher study levels being more affected.^1^ In Africa, a systematic review indicated that about 55.64% of medical students experienced poor sleep quality, a rate significantly higher than the general population.^2^ In Europe, a study involving Italian college students found that inadequate sleep (less than seven hours per night) was common, which negatively impacted their well-being and mental health.^3^

In Saudi Arabia, many studies indicate a high prevalence of poor sleep quality among university students across regions. For example, in the southern region, a study revealed that 64% of undergraduate students experienced poor sleep quality.^4^ In the middle region, a study focusing on medical students found that 75.93% reported poor sleep quality.^5^ In the eastern region, 30.9% had significant sleep quality disorders, and 34.9% of students experienced daytime sleepiness.^6^ These findings reflect the widespread of sleep-related problems among university students in the country.

Smartphone usage, especially at bedtime, significantly affects sleep quality among university students.^7^ In education, smartphones are not merely tools for communication but devices that enhance learning experiences, providing students with access to information, educational apps, and organizational aids.^8^ However, prolonged smartphone use before sleep is associated with difficulty falling asleep, reduced sleep duration, and frequent interruptions during the night.^9^ Activities such as social media, gaming, or watching videos can keep the brain active, making it harder to fall asleep.^10^ In addition, blue light emitted by smartphones can disrupt the sleep cycle.^11^

Poor sleep quality can lead to cognitive deficits, mood disturbances, and decreased academic achievement.^3^ Students who slept poorly, perform worse on exams, solve problems less effectively, and achieve lower academic results.^12^,^13^ Lack of sleep is also linked with deteriorating physical health^14^ irregular exercise^4^ high consumption of caffeine or energy drinks^10^ and unhealthy dietary.^15^ This creates a vicious cycle, where poor sleep exacerbates unbalanced lifestyle habits, further impairing sleep quality. Therefore, this study aims to investigate the influence of smartphones usage habits on sleep quality of undergraduate students in Saudi Arabia. Understanding this relationship can lead to tailored interventions to improve students’ sleep hygiene, enhance their learning capacity, and boost academic performance.

## METHODS

A cross-sectional study was conducted between April and May 2023 at Jazan University, Saudi Arabia. An online questionnaire was constructed using Google Forms and consisted of three sections. The first section covered demographics (e.g., age, gender, marital status, etc.). The second section focused on smartphone usage habits (e.g., average daily use, time spent on smartphone at bedtime, keeping the smartphone close while sleeping, waking up to check the smartphone, etc.). The third section assessed sleep quality using the Pittsburgh Sleep Quality Index (PSQI).^16^,^17^ It includes seven components: subjective sleep quality, sleep latency, sleep duration, habitual sleep efficiency, sleep disturbances, use of sleep medications, and daytime dysfunction. PSQI scores ≥5 indicate disturbed sleep, and scores ≥9 suggest clinical sleep disturbances. In this study, participants were given the option to complete either the English version or the reliable and valid Arabic-translated version of the PSQI survey.^18^ A link to the online questionnaire was posted on social networking platforms such as WhatsApp, Snapchat, Telegram, and X (formerly Twitter). Before completing the questionnaire, participants were informed about the study’s objectives and provided e-consent. Then data was extracted from Microsoft Excel and transferred to Stata/BE 17.0. Frequency, percentages, means and standard deviation (SD) were utilized. Inferential tests including t-test, ANOVA, chi-square test, and multivariable linear regression were also used to assess associations, with significance set at *P* < 0.05.

### Ethical approval:

It was obtained from Jazan University’s research ethics committee (Reference: REC-44/09/606) dated April 06, 2023

## RESULTS

A total of 1,153 participants reported using smartphone at bedtime. The majority was in the 18–24 age range (90.03%), female (82.91%), unmarried (86.21%), and living in villages (63.14%). PSQI global score was 8.19, yielded significantly poor sleep quality. Table-I presents the demographics of the participants in this study.

**Table-I:** Participants’ Characteristics.

Variables	N	(%)
*Demographics*		
*Age*		
18-24	1,038	90.03
> 24	115	9.97
Gender		
Male	197	17.09
Female	956	82.91
*Marital Status*		
Unmarried	994	86.21
Married	159	13.79
*Area of Residence*		
Village	728	63.14
City	425	36.86
*Smartphone Usage Habits*		
*Average Daily Use of Smartphone (hour)*		
≤ 1	18	1.56
> 1 < 4	84	7.29
> 4 < 6	204	17.69
> 6 < 8	285	24.72
> 8	562	48.74
*Time to Use Smartphone*		
Morning	298	25.85
Afternoon	407	35.30
Evening	448	38.86
*Keep Smartphone Close While Sleeping*		
No	88	7.63
Yes	1,065	92.37
*Wake up to Use Smartphone*		
No	640	55.51
Yes	513	44.49
*Wake up to Check Smartphone*		
Once	837	72.59
Twice	187	16.22
Three times or more	129	11.19
*Use Smartphone Immediately when Wake up in the Morning*		
No	177	15.35
Yes	976	84.65
*Time Spent on Smartphone at Bedtime (hour)*		
< 1	353	30.62
1 – 2	518	44.93
> 2	282	24.46
*Outcome*	*Mean*	*SD*
Sleep Quality Score	8.19	3.57

A significant prevalence of smartphone use was found, with 73.46% of participants using their smartphones for more than six hours per day. Nearly three-quarters of the participants reported using their smartphones during the afternoon and evening (74.16%). At bedtime, over two-thirds of participants spent at least one hour on their smartphones (69.38%), and (92.37%) kept their devices close while sleeping. All participants woke up at least once to check their smartphones. The majority (84.65%) used their smartphones immediately when they woke up in the morning.

### Associations between the Sleep Quality Score and Smartphone Usage Habits:

Significant and positive relationships were identified between the sleep quality score and the various smartphone usage habits, as shown in Table-II. The sleep quality score increased significantly as the average number of hours of daily use of a smartphone increased. For example, those who reported more than eight hours of smartphone use per day had a higher sleep quality score (8.55); indicating poorer sleep quality, compared to those who reported an hour or less of smartphone use per day (7.39).

**Table-II:** Comparison of Sleep Quality by Participants’ Smartphone Usage Habits.

Variables	N (%)	Sleep Quality			
		Mean	SD	t-value	p-value
*Smartphone Usage Habits*					
*Average Daily Use of Smartphone (hour)*		*Mean*	*SD*	*F-value*	*p-value*
≤ 1	18 (1.56)	7.39	3.76	5.38	< 0.001 *
> 1 < 4	84 (7.29)	6.86	3.28		
> 4 < 6	204 (17.69)	7.76	3.46		
> 6 < 8	285 (24.72)	8.23	3.55		
> 8	562 (48.74)	8.55	3.60		
*Time to Use Smartphone*		*Mean*	*SD*	*F-value*	*p-value*
Morning	298 (25.85)	8.02	3.45	1.74	0.176
Afternoon	407 (35.30)	8.04	3.69		
Evening	448 (38.86)	8.43	3.54		
*Keep Smartphone Close While Sleeping*					
No	88 (7.63)	7.32	2.97	- 2.38	0.017*
Yes	1,065 (92.37)	8.26	3.61		
*Wake up to Use Smartphone*					
No	640 (55.51)	8.00	3.56	- 1.94	0.052
Yes	513 (44.49)	8.42	3.58		
*Wake up to Check Smartphone*		*Mean*	*SD*	*F-value*	*p-value*
Once	837 (72.59)	7.83	3.51	16.13	< 0.001*
Twice	187 (16.22)	9.02	3.31		
Three times or more	129 (11.19)	9.31	3.88		
*Use Smartphone Immediately when Wake up*					
No	177 (15.35)	7.80	3.53	- 1.58	0.114
Yes	976 (84.65)	8.26	3.57		
*Time Spent on Smartphone at Bedtime (hour)*		*Mean*	*SD*	*F-value*	*p-value*
< 1	353 (30.62)	7.43	3.45	13.03	< 0.001*
1 – 2	518 (44.93)	8.37	3.50		
> 2	282 (24.46)	8.80	3.70		

*Note:* Comparison made using t-test and ANOVA,

* *p* < 0.05.

Using smartphones in the evening had a slightly greater impact on the sleep quality score as opposed to using them in the morning or afternoon. In addition, participants who stated keeping their smartphone close to them while they were sleeping had considerably greater sleep quality scores than those who did not. Similarly, participants who reported checking their smartphones three or more times during the night had significantly higher sleep quality scores than those who only checked them once, reflecting poorer sleep quality.

### The Most Prevalent Sleep Issues Among Smartphone Users at Bedtime:

***T***he most prevalent sleep issues among three groups of participants in our sample. Group-I, who used their smartphones for less than one hour before bed; Group-II, who used their smartphones for 1-2 hours before bed; and Group-III, who used smartphones for more than two hours before bedtime is highlighted in Table-III.

**Table-III:** Components of PSQI among the Bedtime Smartphone Users Groups.

PSQI Components	Time Spent on Smartphone at Bedtime			*P*-value
	Group-I (< 1 hr.) (%)	Group-II (1 to 2 hrs.) (%)	Group-III (> 2 hrs.) (%)	
*Subjective sleep quality*				
Very good	33.99	24.32	25.18	< 0.001 *
Fairly good	45.04	49.81	39.72	
Fairly bad	15.58	17.95	23.05	
Very bad	5.38	7.92	12.06	
*Sleep latency (minute)*				
< 15	16.15	10.42	12.41	< 0.001 *
16-30	36.54	27.03	24.11	
31-60	31.44	34.36	32.27	
> 60	15.86	28.19	31.21	
*Sleep duration (hour)*				
> 7	38.81	36.49	41.84	0.143
6-7	15.86	15.44	9.57	
5-6	30.03	34.17	31.21	
< 5	15.30	13.90	17.38	
*Habitual Sleep efficiency*				
≥ 85%	52.12	53.28	45.39	0.215
75-84%	9.07	8.69	8.87	
65-74%	9.92	8.11	7.80	
< 65%	28.90	29.92	37.94	
*Sleep disturbance*				
Not during past month	10.20	6.37	12.41	< 0.001*
Less than once a week	60.62	55.79	41.84	
Once or twice a week	26.35	35.14	40.78	
Three or more times a week	2.83	2.70	4.96	
*Use of sleep medication*				
Not during past month	88.10	80.12	75.53	0.001 *
Less than once a week	5.38	10.81	14.89	
Once or twice a week	4.53	5.79	4.61	
Three or more times a week	1.98	3.28	4.96	
*Daytime dysfunction*				
No problem at all	22.38	16.41	23.40	0.004 *
Only a very slight problem	40.79	38.42	33.69	
Somewhat of a problem	27.20	33.40	26.24	
A very big problem	9.63	11.78	16.67	
*PSQI Score Mean (SD)*	7.43 (3.45)	8.37 (3.50)	8.80 (3.70)	

The first and most significant issue was increased sleep latency, with 31.21% of participants taking more than 60 minutes to fall asleep. Sleep latency was nearly twice as high in Group-III compared to Group-I (15.86%). The second issue was a significant daytime dysfunction. It was reported by 16.67% of participants in Group-III compared to 9.63% by Group-I, indicating considerable impairment in daily functioning due to poor sleep quality. This was followed by a high prevalence of sleep disturbances, with 4.96% experiencing disruptions three or more times a week. Group-III (4.96%) experienced almost twice as many weekly sleep disruptions as Group-I (2.83%). Likewise, the use of sleep medications was another major issue, with 4.96% of participants relying on them three or more times a week. The frequency of taking sleep medications per week was approximately three times higher in Group-III (4.96%) than in Group-I (1.98%).

**Fig.1:**
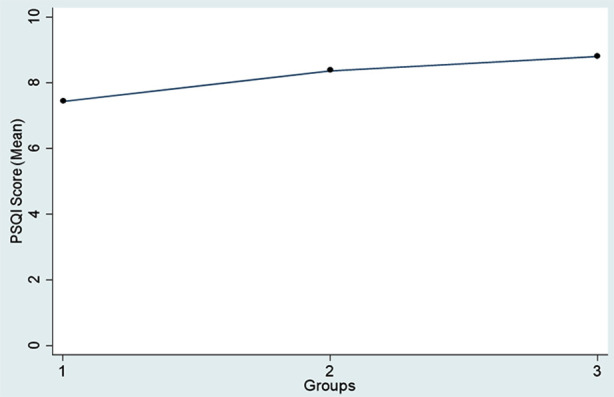
Comparison of Pittsburgh Sleep Quality Index Scores among the Bedtime Smartphone Users’ Groups.

Regression analysis revealed that increased smartphone usage before bedtime is associated with poorer sleep quality, as reflected in higher PSQI scores. Group-III had the highest average PSQI score of 8.80, indicating the poorest sleep quality among the groups, as shown in Fig.1. Group-II had a moderate average PSQI score of 8.37, reflecting a decline in sleep quality compared to the first group. In contrast, Group-I had the lowest average PSQI score of 7.43, indicating the best sleep quality. Furthermore, Group-III reported having a very bad sleep quality (12.06%) as compared to Group-I (5.38%).

### Key Predictors of Poor Sleep Quality Among Smartphone Users at Bedtime:

The multivariable linear regression analysis revealed the three key predictors that were significantly associated with poorer sleep quality, as presented in Table-IV. These predictors were: using a smartphone in the evening, waking up multiple times to check the smartphone, and spending 1-2 hours on a smartphone at bedtime. These habits were linked to higher sleep disturbance scores, indicating a detrimental effect on sleep quality.

**Table-IV:** The Multivariable Linear Regression Analysis of Individual Factors Predicting Sleep Quality Score.

Variables	Coefficient (β)	SE	t-value	*p*-value
*Smartphone Usage Habits*				
*Average Daily Use of Smartphone (hour)*				
≤ 1	Ref			
> 1 < 4	- 0.67	0.909	- 0.74	0.461
> 4 < 6	0.15	0.862	0.17	0.861
> 6 < 8	0.45	0.854	0.53	0.596
> 8	0.54	0.844	0.64	0.524
*Time to Use Smartphone*				
Morning	Ref			
Afternoon	0.21	0.272	0.78	0.435
Evening	0.54	0.268	2.02	0.044 *
*Keep Smartphone Close While Sleeping*				
No	Ref			
Yes	0.65	0.395	1.64	0.102
*Wake up to Use Smartphone*				
No	Ref			
Yes	- 0.32	0.245	- 1.30	0.192
*Wake up to Check Smartphone*				
Once	Ref			
Twice	1.09	0.301	3.63	< 0.001 *
Three times or more	1.33	0.375	3.55	< 0.001 *
*Use Smartphone Immediately when Wake up*				
No	Ref			
Yes	0.07	0.305	0.22	0.824
*Time Spent on Smartphone at Bedtime (hour)*				
< 1	Ref			
1 – 2	0.64	0.249	2.58	0.010 *
> 2	0.83	0 .309	2.68	0.007 *

*SE*= Standard Error; REF= Reference Group,

* P < 0.05.

Based on the regression analysis, the factor that produced the poorest quality of sleep was waking up to check smartphones twice or more. Compared to those reported waking up once to check their smartphones, the sleep quality score increased by 1.09 and 1.33 points for those reported waking up twice or three time or more, respectively, (all *p’*s <0.001). Similarly, the sleep quality score was 0.64 and 0.83 points higher among those spent 1-2 hours (*p*=0.010) or more than two hours (*p*=0.007) on their smartphones at bedtime, respectively, compared to those spent less than one hour. Finally, compared to those reported using their smartphone in the morning, the sleep quality score increased by 0.54 points for those reported using it in the evening (*p*=0.044).

## DISCUSSION

Smartphone usage habits are key predictors of poor sleep among undergraduate students. In this study, the average PSQI score was 8.19, indicating significantly disturbed sleep quality, with many participants using their devices for more than two hours at bedtime. These findings are consistent with previous research showing that extensive smartphone use before bed adversely affects sleep, often resulting in PSQI scores exceeding five.^1^,^10^,^19^

In this study, all participants woke up at least once to check their smartphones, and this behaviour had the most significant negative impact on sleep quality. Regression analysis also revealed issues like prolonged sleep latency, frequent disturbances, reliance on sleep medications, and daytime dysfunction among those with extensive smartphone use before bedtime. Similar studies suggest that the presence of smartphones in the bedroom contributes to stress and anxiety due to notifications, calls and text messages, and the pressure to stay connected.^3^,^20^,^21^

Despite the severe consequences of prolonged bedtime smartphone use, limited research addresses solutions for these sleep issues among university students.^22^,^23^ Thus, more studies are needed to develop effective interventions to mitigate the negative impacts of smartphone use on sleep health among university students. Creating a smartphone-free bedroom environment can be one of the effective strategies to enhance sleep hygiene.^24^ By eliminating screens from the bedroom, students can maintain natural melatonin production, promoting a more natural sleep cycle.^25^,^26^ Furthermore, a smartphone-free bedroom contributes to better cognitive functions and academic performance by ensuring students are well-rested and maintaining consistent bedtime routines.^2^,^27^

### Limitations:

The strengths of this study include the large sample size, the use of validated and reliable scales, and exploring various factors associated with sleep quality. However, a limitation is that the data relied on self-reported information from participants.

## CONCLUSION

High smartphones use negatively impacts sleep quality. Educating students on its effects, especially at bedtime, is essential. Future research should focus on strategies to encourage technology-free bedrooms that foster a relaxing environment and consistent bedtime routines like reading or meditating.

### Authors’ Contribution:

**MA & MAM:** Acquisition of data. **AMA:** Software and data analysis. **AMA & MA:** Interpretation of data.

All authors are conceived, designed, and drafted the manuscript and approved the final version. They are also accountable for the integrity of the study.

## References

[1] Hu B, Shen W, Wang Y, Wu Q, Li J, Xu X (2024). Prevalence and related factors of sleep quality among Chinese undergraduates in Jiangsu Province:multiple models'analysis. Front. psychol.

[2] Nakie G, Takelle GM, Rtbey G, Andualem F, Tinsae T, Kassa MA (2024). Sleep quality and associated factors among university students in Africa:a systematic review and meta-analysis study. Front Psychiatry.

[3] Dagani J, Buizza C, Cela H, Sbravati G, Rainieri G, Ghilardi A (2024). The interplay of sleep quality, mental health, and sociodemographic and clinical factors among Italian college freshmen. J clin Med.

[4] Mahfouz MS, Ali SA, Bahari AY, Ajeebi RE, Sabei HJ, Somaily SY (2020). Association Between Sleep Quality and Physical Activity in Saudi Arabian University Students. Nat Sci Sleep.

[5] Alhusseini NK, Ramadan M, Almasry Y, Atout M, Hamsho K, Mahmoud M (2022). Effects of sleep quality on academic performance and psychological distress among medical students in Saudi Arabia. Health Scope.

[6] Zafar M, Omer EO, Elfatih M, Ansari K, Kareem A, Fawaz A (2020). Daytime sleepiness and sleep quality among undergraduate medical students in Dammam, Saudi Arabia. Indian J Med Specialities.

[7] Fook CY, Narasuman S, Abdul Aziz N, Tau Han C (2021). Smartphone usage among university students. Asian J Univ Educ.

[8] Alfawareh HM, Jusoh S (2014). Smartphones usage among university students:Najran University case. Int J Acad Res.

[9] Yang J, Fu X, Liao X, Li Y (2020). Association of problematic smartphone use with poor sleep quality, depression, and anxiety:A systematic review and meta-analysis. Psychiatry Res.

[10] Elsheikh AA, Elsharkawy SA, Ahmed DS Impact of smartphone use at bedtime on sleep quality and academic activities among medical students at Al-Azhar University at Cairo. J Public Health.

[11] Randjelović P, Stojanović N, Ilić I, Vučković D (2023). The effect of reducing blue light from smartphone screen on subjective quality of sleep among students. Chronobiol Int.

[12] Owusu-Marfo J, Lulin Z, Antwi HA, Kissi J, Antwi MO, Asare IO (2018). The Effect of Smart mobile devices usage on Sleep Quality and academic performance –A Narrative Review. Can J Appl Sci Technol.

[13] Seoane HA, Moschetto L, Orliacq F, Orliacq J, Serrano E, Cazenave MI (2020). Sleep disruption in medicine students and its relationship with impaired academic performance:a systematic review and meta-analysis. Sleep Med. Rev.

[14] Wang F, Boros S (2021). The effect of physical activity on sleep quality:a systematic review. Eur J Physiother.

[15] Ramón-Arbués E, Granada-López JM, Martínez-Abadía B, Echániz-Serrano E, Antón-Solanas I, Jerue BA (2022). The Association between Diet and Sleep Quality among Spanish University Students. Nutrients.

[16] Buysse DJ, Reynolds CF, Monk TH, Berman SR, Kupfer DJ (1989). The Pittsburgh sleep quality index:A new instrument for psychiatric practice and research. Psychiatry Res.

[17] Hinz A, Glaesmer H, Brähler E, Löffler M, Engel C, Enzenbach C (2017). Sleep quality in the general population:psychometric properties of the Pittsburgh Sleep Quality Index, derived from a German community sample of 9284 people. Sleep Med.

[18] Suleiman KH, Yates BC, Berger AM, Pozehl B, Meza J (2010). Translating the Pittsburgh sleep quality index into Arabic. Western J Nurs Res.

[19] Ozcan B, Acimis NM (2021). Sleep Quality in Pamukkale University Students and its relationship with smartphone addiction. Pak J Med Sci.

[20] Irshad K, Ashraf I, Azam F, Shaheen A (2022). Burnout prevalence and associated factors in medical students in integrated modular curriculum:A cross-sectional study. Pak J Med Sci.

[21] Alzhrani AM, Aboalshamat KT, Badawoud AM, Abdouh IM, Badri HM, Quronfulah BS (2023). The association between smartphone use and sleep quality, psychological distress, and loneliness among health care students and workers in Saudi Arabia. PLoS One.

[22] Exelmans L, Van den Bulck J (2016). Bedtime mobile phone use and sleep in adults. Soc Sci Med.

[23] Schlarb AA, Friedrich A, Claßen M Sleep problems in university students–an intervention. Neuropsychiatr Dis Treat.

[24] Kheirinejad S, Visuri A, Ferreira D, Hosio S (2023). “Leave your smartphone out of bed”:quantitative analysis of smartphone use effect on sleep quality. Pers Ubiquitous Comput.

[25] Chang AM, Aeschbach D, Duffy JF, Czeisler CA (2015). Evening use of light-emitting eReaders negatively affects sleep, circadian timing, and next-morning alertness. Proc Natl Acad Sci U S A.

[26] Higuchi S, Motohashi Y, Liu Y, Ahara M, Kaneko Y (2003). Effects of VDT tasks with a bright display at night on melatonin, core temperature, heart rate, and sleepiness. J Appl Physiol (1985).

[27] Schmickler JM, Blaschke S, Robbins R, Mess F (2023). Determinants of sleep quality:a cross-sectional study in university students. Int J Environ Res Public Health.

